# Supplementary material for: KCNN4 Promotes the Stemness Potentials of Liver Cancer Stem Cells by Enhancing Glucose Metabolism
Source: Int J Mol Sci. 2022 Jun 23;23(13):6958. doi: 10.3390/ijms23136958 (PMC9266406; doi:10.3390/ijms23136958)
Supplement: Supplementary file 1 [file ijms-23-06958-s001.zip › ijms-1729822-supplementary/supplementary figure.pdf]

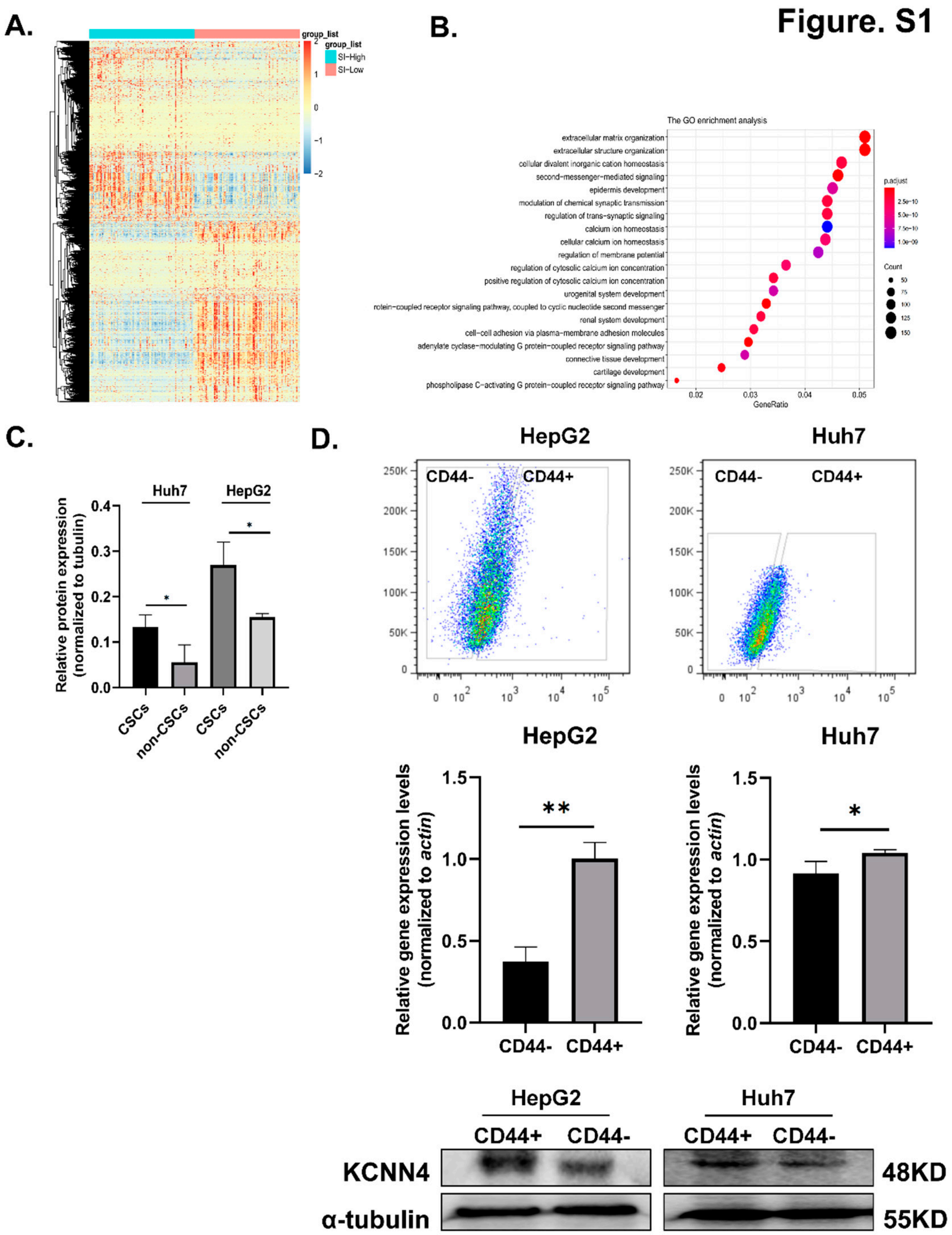

Supplementary Figure S1.

(A) Heat maps of different genes. (B) was GO enrichment analysis of different genes. (C) was *KCNN4* protein level in stem cells and non-stem cells in HepG2 and Huh7 cell lines. (D) was the schematic diagram of FACS in HepG2 and Huh7 cells, the relative expression of mRNA and protein of *KCNN4* were shown as blow.

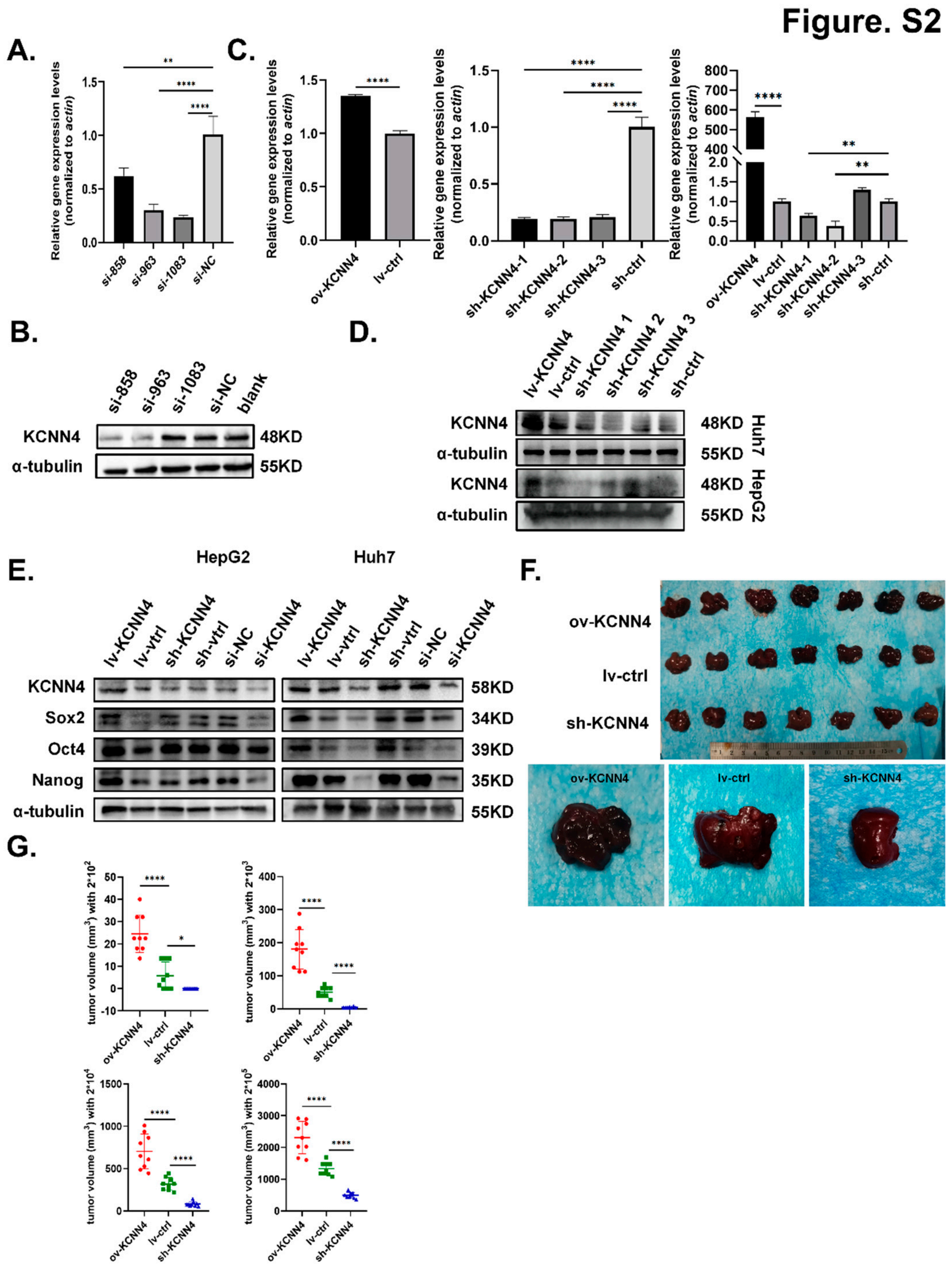

Supplementary Figure S2.

(A, B) The efficacies of knockdown of *KCNN4* by siRNA were examined by qPCR(A) and western blot(B) in HCC cells. (C, D) The efficacies of knockdown or overexpression of *KCNN4* by lentiviruses were examined by qPCR(C) and western blot(D) in HCC cells. ov-*KCNN4*, overexpression of *KCNN4*. lv-ctrl, the control of gene overexpression. sh-*KCNN4*, *KCNN4* interference. sh-ctrl, the control of RNA interference. (E) The expression of stem cell transcription factors, including Sox2, Oct4, and Nanog in HepG2 and Huh7. The liver of tumor-bearing mice was dissected and the tumor formation was observed (F, above) The typical pictures of each group was shown (F, below). (G) In the gradient dilution model of

the NOD-SCID mouse, tumor volume(mm^3) was shown.

Figure. S3

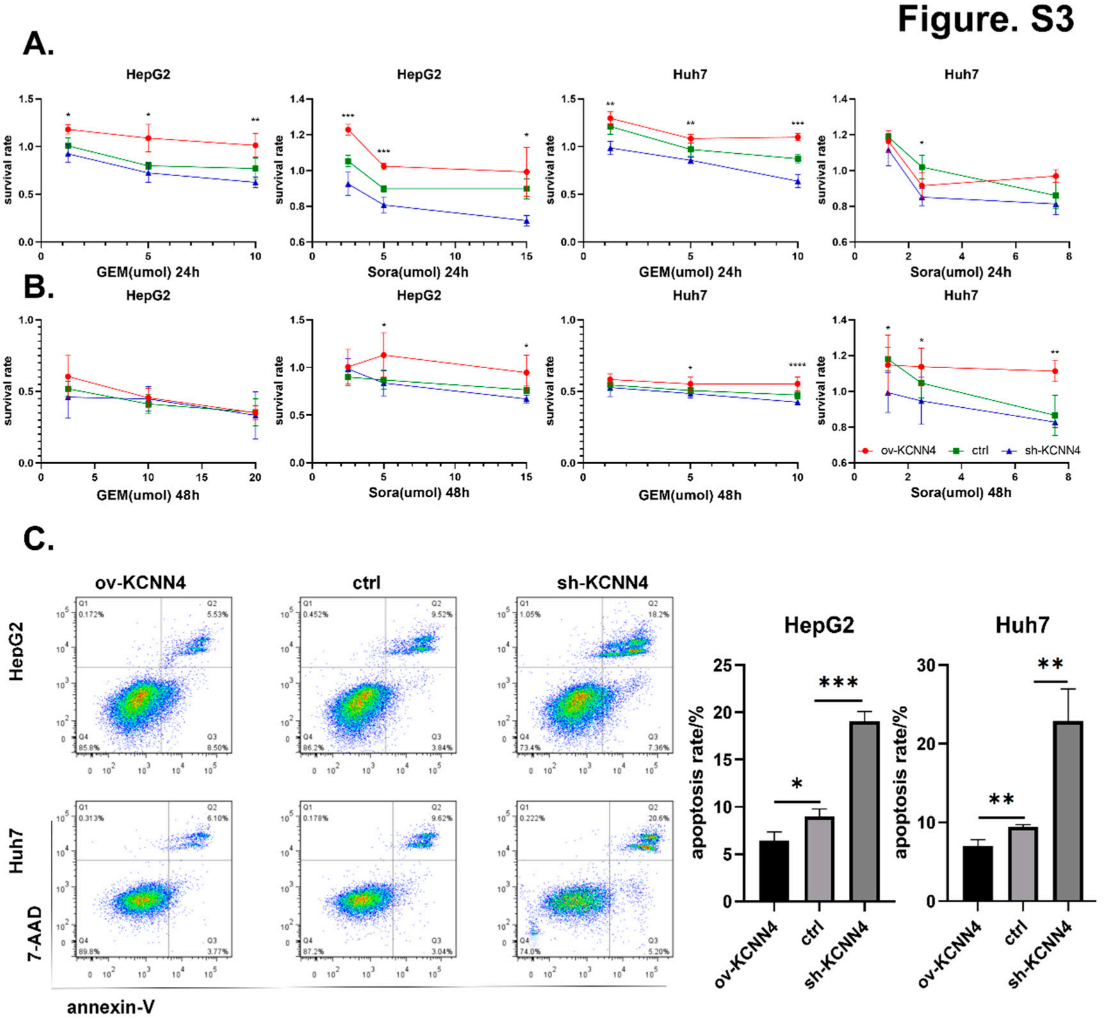

Supplementary Figure S3. ***KCNN4*** **enhanced the chemotherapy resistance of HCC.**

Cell proliferation was detected using the CCK8 assay. HepG2 and Huh7 cells (2×10^3 cells/well) were seeded in 96-well plated and treated with sorafenib and the gemcitabine in HepG2/Huh7 and the absorbances were measured at 450 nm in 24h (A) and 48h (B), respectively. (C) Drug-induced apoptosis was detected by flow cytometry treated with sorafenib (10umol in HepG2 and 7.5umol in Huh7) after 48h.

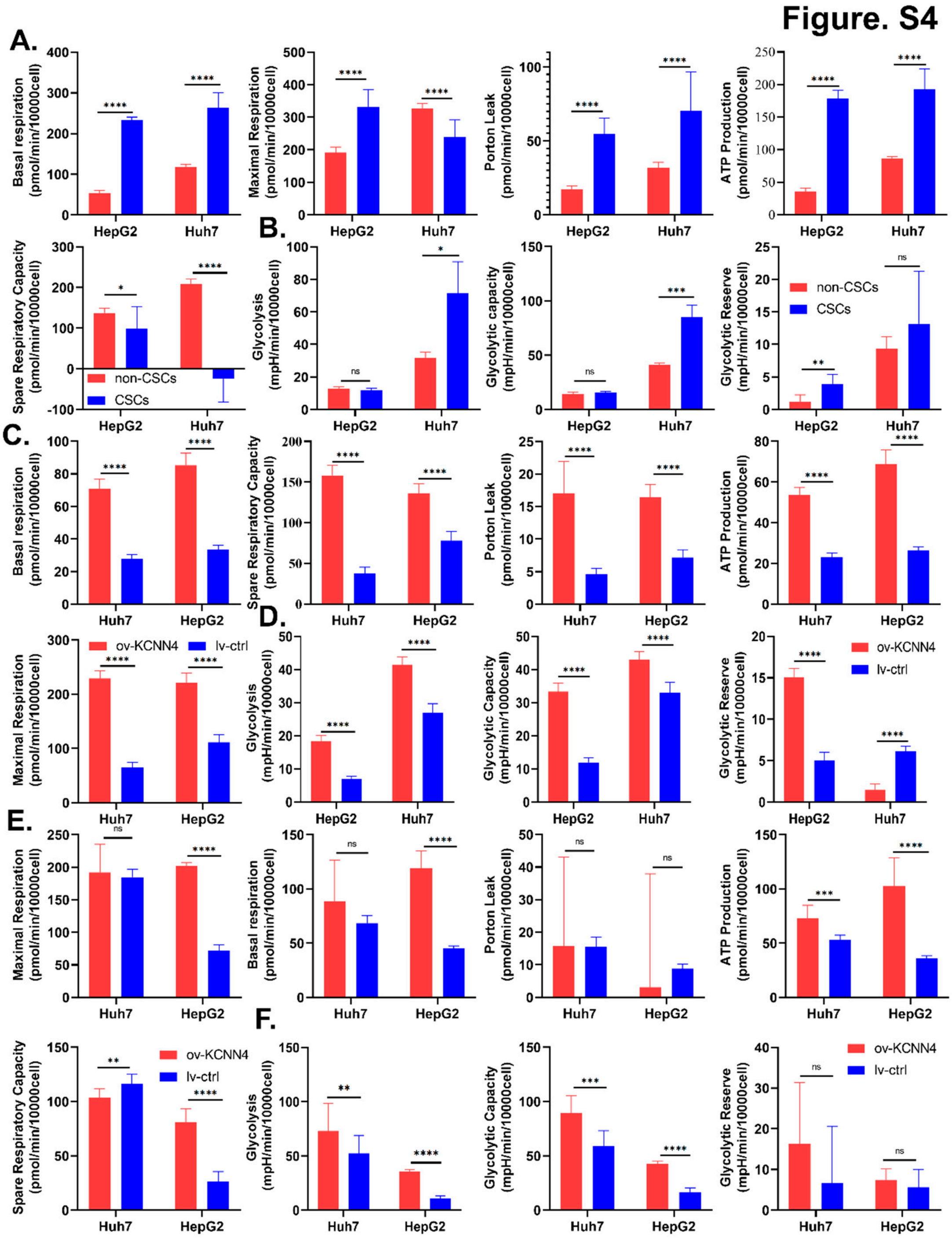

Supplementary Figure S4. ***KCNN4*** **enhances metabolic fitness in LCSCs by upregulating glucose metabolism.**

Mito-stress test (A, C, E) and glycolysis test (B, D, F) were executed for OCR (Oxygen Consumption Rate) and ECAR (Extracellular Acidification Rate) in adherent cells and stem cells. (A) adherent cells vs stem cells. Mito-stress test. The bar charts represented basal respiration, maximal respiration, proton leak, ATP production, and spare respiration capacity, respectively. (B) adherent cells vs stem cells. glycolysis-stress test. The bar charts represented glycolysis, glycolytic capacity, and glycolytic reserve, respectively. (C) ov-*KCNN4* vs lv-*ctrl* in HepG2 and Huh7 cell lines. Mito-stress test. The bar charts represented basal

respiration, maximal respiration, proton leak, ATP production, and spare respiration capacity, respectively. (D) ov-*KCNN4* vs lv-*ctrl* in HepG2 and Huh7 cell lines. glycolysis-stress test. The bar charts represented glycolysis, glycolytic capacity, and glycolytic reserve, respectively. (E) ov-*KCNN4* vs lv-*ctrl* in stem cells enriched from the sphere of HepG2 and Huh7 cell lines. Mito-stress test. The bar charts represented basal respiration, maximal respiration, proton leak, ATP production, and spare respiration capacity, respectively. (F) ov-*KCNN4* vs lv-*ctrl* in stem cells enriched from the sphere of HepG2 and Huh7 cell lines. glycolysis-stress test. The bar charts represented glycolysis, glycolytic capacity, and glycolytic reserve, respectively. These data represent the mean ±SD from at least three independent experiments. 2-way Student's t-test. * $p < 0.05$, **$p < 0.01$, ***$p<0.001$, ****$p<0.0001$.

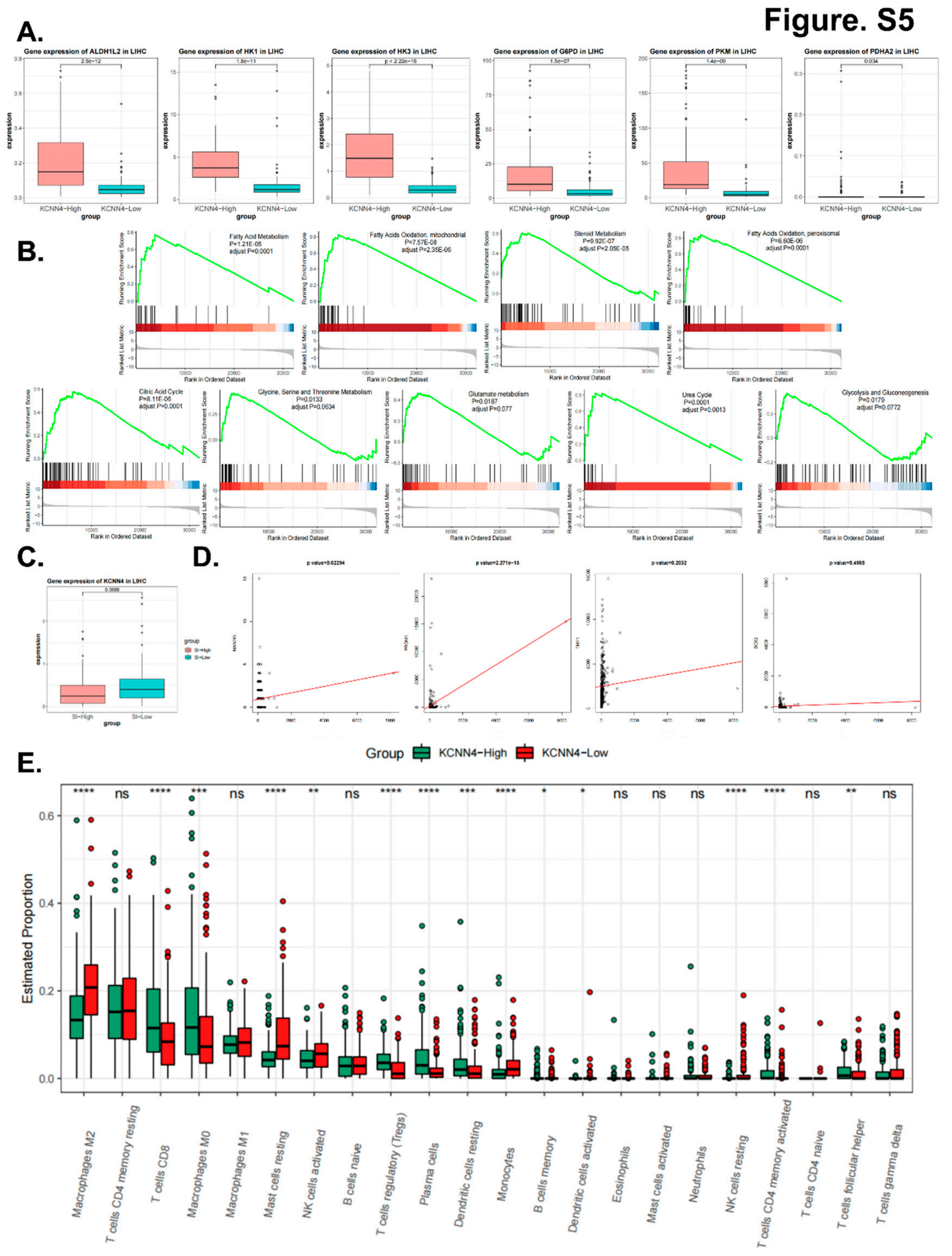

Supplementary Figure S5. *KCNN4* enhanced metabolic fitness by upregulating energy metabolism. The different expressions of key enzymes were shown as (A). According to the expression of *KCNN4*, the metabolism-related pathways were enriched as (B). (C) was the expression level of *KCNN4* in SI-HIGH and SI-LOW groups, respectively. (D) were the correlations between *KCNN4* and stemness transcription factors. (E) were the correlations between expression of *KCNN4* and immune infiltration of 22 immune cell types in patients with HCC.

Figure. S6

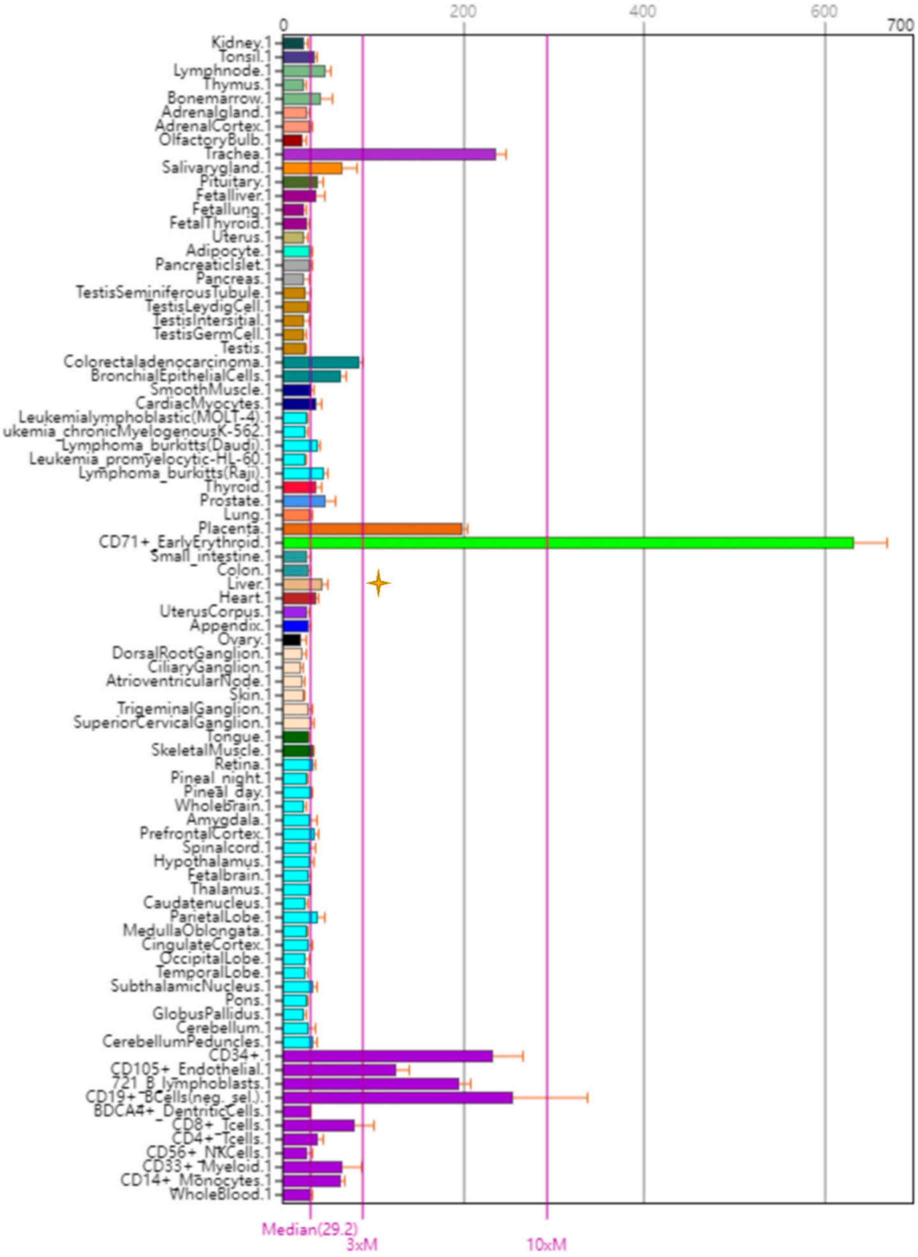

Supplementary Figure S6. The tissue-specific pattern of mRNA expression of *KCNN4* in BioGPS database.
