# Supplementary material for: KCNN4 Promotes the Stemness Potentials of Liver Cancer Stem Cells by Enhancing Glucose Metabolism
Source: Int J Mol Sci. 2022 Jun 23;23(13):6958. doi: 10.3390/ijms23136958 (PMC9266406; doi:10.3390/ijms23136958)
Supplement: Supplementary file 1 [file ijms-23-06958-s001.zip › ijms-1729822-supplementary/supplementary table.pdf]

Supplementary Table S1. ***KCNN4*** **promoted** ***in vivo*** **tumor formation ability by increasing stem cell potentials.**

	**Tumor weight (g)**			**Tumor volume (mm³)**			**Median tumor incidence time (d)**		
	ov-KCNN4	lv-ctrl	sh-KCNN4	ov-KCNN4	lv-ctrl	sh-KCNN4	ov-KCNN4	lv-ctrl	sh-KCNN4
**$2*10^2$**	0.026±0.008	0.010±0.006	0.000±0.000	24.560±8.443	5.778±6.129	0.000±0.000	60±4	70±4	75±0
**$2*10^3$**	0.021±0.082	0.059±0.014	0.004±0.001	180.170±59.800	50.830±15.490	4.857±1.574	53±1	55±1	71±2
**$2*10^4$**	0.761±0.239	0.372±0.108	0.072±0.027	702.600±205.900	318.300±75.480	82.830±27.680	36±2	42±2	54±1
**$2*10^5$**	2.413±0.573	7.389±0.214	0.515±0.089	2309.000±506.400	1329.000±202.500	491.800±90.210	25±3	32±1	43±1

Supplementary Table S2. ***KCNN4*** **promoted** ***in vivo*** **tumor formation ability by enhancing CSC frequency.**

***No.* of injected cells** / **Groups**	**$2*10^5$**	**$2*10^4$**	**$2*10^3$**	**$2*10^2$**	**CSC frequency**	***P*-values**
**(A) ov-KCNN4**	9/9	9/9	9/9	9/9	1/1	**A *vs.* B:** 0.029
**(B) lv-ctrl**	9/9	9/9	9/9	6/9	1/182	**A *vs.* C:** 2.31e-08
**(C) sh-KCNN4**	9/9	9/9	7/9	0/9	1/1629	**B *vs.* C:** 0.000256

Supplementary Table S3. Formulation of sphere culture medium.

supplementary	concentration	Manufacture (Catalog)
DMEM/F12 base medium	100ml	Thermo (A4192001)
Penicillin-Streptomycin	100IU/ml Penicillin and 100ug/ml Streptomycin	Corning® (30-002-CI)
L-glutamine	2mm/l	Corning® (25-005-CI)
Non-Essential Amino Acids Solution (100X)	1ml	Thermo (11140050)
B-27 supplement (50X), serum-free	2ml	Gibco (17504-044)
2-mercaptoethanol	75uM	Gibco (21985023)
N-2 supplementary liquid (100X)	1ml	Gibco (17502001)
EGF	20ng/ml	Sigma (#E9644)
Human Recombinant IGF-I, ACF	5U/L	Stem Cell (#78142)
Heparin solution	0.96IU/ml	Stem Cell (#07980)
15% glucose	4ml	Sigma(G7021)

Supplementary Table S4. sequence of primer, siRNA, and lentivirus

Gene	primers	sequence	
		**Forward** (5' to 3')	**Reverse** (3' to 5')
**Stemness transcription factor**	CD133	TGGATGCAGACCTTGACAACGT	ATACCTGCTACGACAGTCGTGGT
	CD90	GACCCGTGAGACAAAGAAGC	GCCCTCACACTTGACCAGTT
	Bmi1	TGGAGAAGGAATGGTCCACTTC	GTGAGGAAACTGTGGATGAGGA
	Oct4	CTTGCTGCAGAAGTGGGTGGAGGAA	CTGCAGTGTGGGTTTCGGGCA
	Sox2	GCCGAGTGGAAACTTTTGTCG	GGCAGCGTGTACTTATCCTTCT
	Nanog	AATACCTCAGCCTCCAGCAGATG	TGCGTCACACCATTGCTATTCTTC
	β-actin	CCTGGCACCCAGCACAAT	GGGCCGGACTCGTCATAC
**KCNN4**	KCNN4-1	TGCTCAACGCTTCCTACCGCAGCAT	CTCTCGGCCACGGACAGCACCCA
	KCNN4-2	TAACAAGGCAGAGAAGCACG	CCTTCCTGCGAGTATGTTTGTA
**TCA**	PDHK	TTGGCTGGATTTGGTTATGGT	CTTCAGGCGTGGTCTTGTAATG
	PDHA	AACCCCACAGACCATCTCATCA	CCTTTCCCTTTAGCACAACCTC
	PDHB	TGGAGAAGAAGTTGCCCAGTATG	ACCAGCCATAGCTGCACCTA
**Glycolysis**	LDHA	GATTCAGCCCGATTCCGTTAC	GAGTCCAATAGCCCAGGATGTG
	GLUT1	TCACTGTGCTCCTGGTTCTGTTC	GCTCCTCGGGTGTCTTGTCA
	HK2	GCCCGCCAGAAGACATTAGA	CCTTGCTCAGACCTCGCTCC
	PFK1	GGAGGAACACCTTTGTCGCC	GTCAAAGGCTGATGGCGTCC
	PKM	GCTTCTGACCCCATCCTCTACC	CGTTATCCAGCGTGATTTTGAG
**Mitochondrial biosynthesis**	TFAM	TAGGGCGGAGTGGCAGGTA	TTCTTCCCAAGACTTCATTTCATTAT
	PGC1α	AAAGGATGCGCTCTCGTTCA	CTTCAGCCTCTCGTGCTGAT
	NRF1	CAGGCGGTGGCATCGTT	GCGGTTTCCCCAGACAAGAC

**siRNA-seq (Genephrama)**	si-KCNN4-858(5' to 3')	GGAAGCUGGAGUUUAACAATT
		UUGUUAAACUCCAGCUUCCTT
	si-KCNN4-1083(5' to 3')	GGGAACAAGUGAACUCCAUTT
		AUGGAGUUCACUUGUUCCCTT
	si-KCNN4-963(5' to 3')	CCUGGAUGUUCUACAAACATT
		UGUUUGUAGAACAUCCAGGTT
	negative control (5' to 3')	UUCUCCGAACGUGUCACGUTT
		ACGUGACACGUUCGGAGCATT

**lentivirus**	ov-KCNN4(PCR primers of target gene)	AGGTCGACTCTAGAGATCCCGCCACCATGGGCGGGGATCTGGTG
		CTTCCATGGTGGCGACCGGCTTGGACTGCTGGCTGGGTTCTG
	lv-ctrl	TTCTCCGAACGTGTCACGT
	sh-KCNN4-1	CAAGATGCACATGATCCTGTA
	sh-KCNN4-2	GCCTGGATGTTCTACAAACAT
	sh-KCNN4-3	CGCTCTCAATCAAGTCCGCTT
	sh-ctrl	TTCTCCGAACGTGTCACGT
